# Supplementary material for: Proteomic and Metabolomic Evaluation of Insect- and Herbicide-Resistant Maize Seeds
Source: Metabolites. 2022 Nov 7;12(11):1078. doi: 10.3390/metabo12111078 (PMC9696663; doi:10.3390/metabo12111078)
Supplement: Supplementary file 1 [file metabolites-12-01078-s001.zip › metabolites-1989896-supplementary.pdf]

metabolites

MDPI

Article

Proteomic and metabolomic evaluation of insect- and herbicide-resistant maize seeds

Weixiao Liu, Lixia Meng, Weiling Zhao, Zhanchao Wang, Chaohua Miao, Yusong Wan and Wujun Jin*

Biotechnology Research Institute, Chinese Academy of Agricultural Sciences, Beijing 100081, China
* Correspondence: jinwujun@caas.cn

Materials and methods

1. Protein preparation and trypsin digestion

Maize seeds were ground in liquid nitrogen and incubated in lysis buffer (Genecreate, Wuhan, China) (7 M of urea, 2 M of thiourea, 4% SDS, and 40 mM of Tris-HCl (pH 8.5)) containing 1 mM of PMSF and 2 mM of EDTA (final concentration) for 5 min; then, 10 mM of DTT (Sigma, Shanghai, China) (final concentration) was added to the sample. The suspension was sonicated for 10 min on ice and then centrifuged at 4 °C and 13,000 rpm for 20 min. The supernatant was mixed with 4 volumes of pre-cooled acetone at −20 °C for 2 h. After centrifugation, the protein pellets were air-dried and resuspended in 8 M of urea/100 mM of TEAB (Sigma, Shanghai, China). The protein samples were reduced with 10 mM of DTT at 56 °C for 30 min and then alkylated with 50 mM of iodoacetamide (IAM) (Sigma, Shanghai, China) at room temperature for 30 min in the dark. Next, 4 volumes of pre-cooled acetone were added at −20 °C for 2 h. After centrifugation, the protein pellets were air-dried and resuspended in 8 M of urea (Sigma, Shanghai, China) /100 mM of TEAB (pH 8.0). Then, the total protein concentration was measured using the Bradford method.

Trypsin (MS grade, Sigma, Shanghai, China) and 100 μg of protein from each sample were mixed at an enzyme–protein ratio of 1:50 (w/w). Digestion was performed at 37 °C for 16 h. After digestion, the peptides were desalted using C18 columns and dried with a vacuum concentration.

2. LC–MS/MS analysis

The dried peptide sample was reconstituted with a 0.1% formic acid (FA) (HPLC grade, Sigma, Shanghai, China) aqueous solution and then centrifuged at 15,000 rpm for 10 min. Then, the sample solution was analyzed by HPLC–MS. The peptides were dissolved in mobile phase A (0.1% FA aqueous solution) and then separated using EASY-nLC 1200 ultra-high performance liquid system (Thermo Fisher Scientific, Waltham, MA, USA). The peptides were bound to the C18 capture column and the analytical column (Eksigent, San Francisco, CA, USA), then separated using a Q-Exactive HF (Thermo Fisher Scientific, Waltham, MA, USA) equipped with an EASY-Spray nanoliter electric spray ion source. Two mobile phases (A: 0.1% FA; B: 80% acetonitrile (ACN) (HPLC grade, Sigma, Shanghai, China), 0.1% FA) were used to establish analytical gradients over 60, 90, and 120 min. The flow rate of the liquid phase was set to 300 nL/min. After separation from the nanoliter liquid phase, the peptides were ionized by a nanoliter electric spray ion source, which could convert the ions from the solution into gas-phase ions. The gas-phase ions entered the Q-Exactive HF, and the exact mass of the parent ions and the information on the fragment ions were used to determine the peptide sequence.

3. UPLC Conditions and ESI-Q TRAP-MS/MS

The extracted metabolites were analyzed using an UPLC–ESI-MS/MS system (UPLC, Shim-pack UFLC SHIMADZU CBM30A system; MS, Applied Biosystems 4500 Q TRAP). The analytical conditions were as follows: UPLC: column, Agilent SB-C18 (1.8 μm, 2.1 mm × 100 mm); mobile phase consisting of solvent A (98% DDH_2O, 0.1% FA) and solvent B (ACN). Sample measurements were performed with a gradient program that employed the starting conditions of 95% A and 5% B. Within 9 min, a linear gradient to 5% A and 95% B was programmed, and a composition of 5% A and 95% B was maintained for 1 min. Subsequently, the composition was adjusted to 95% A and 5.0% B within 1.10 min and maintained for 2.9 min. The column oven was set to 40 °C, while the injection volume was 4 μL. The effluent was alternatively connected to an ESI-triple quadrupole-linear ion trap (QTRAP)-M.

The ESI source operation parameters were as follows: ion source and turbo spray; source temperature of 550 ℃; ion spray voltage (IS) of 5500 V (positive ion mode)/−4500 V (negative ion mode); ion source gas I (GSI), gas II (GSII), and curtain gas (CUR) set at 50, 60, and 30.0 psi, respectively; high collision gas (CAD). Instrument tuning and mass calibration were performed with 10 and 100 μmol/L polypropylene glycol solutions in QQQ and LIT modes, respectively. QQQ scans were acquired as MRM experiments with the collision gas (nitrogen) set to 5 psi. The DP and CE for individual MRM transitions were carried out with further DP and CE optimization. A specific set of MRM transitions were monitored for each period according to the metabolites eluted within this period.

4. Data analysis

MaxQuant 1.6.17.0 was used to retrieve and analyze the mass spectrometry data. Protein identification was performed against the UniProt *Zea mays* (maize) database supplemented with four foreign proteins, namely, EPSPS, Cry1Ab, Cry3Bb, and Cry1F.

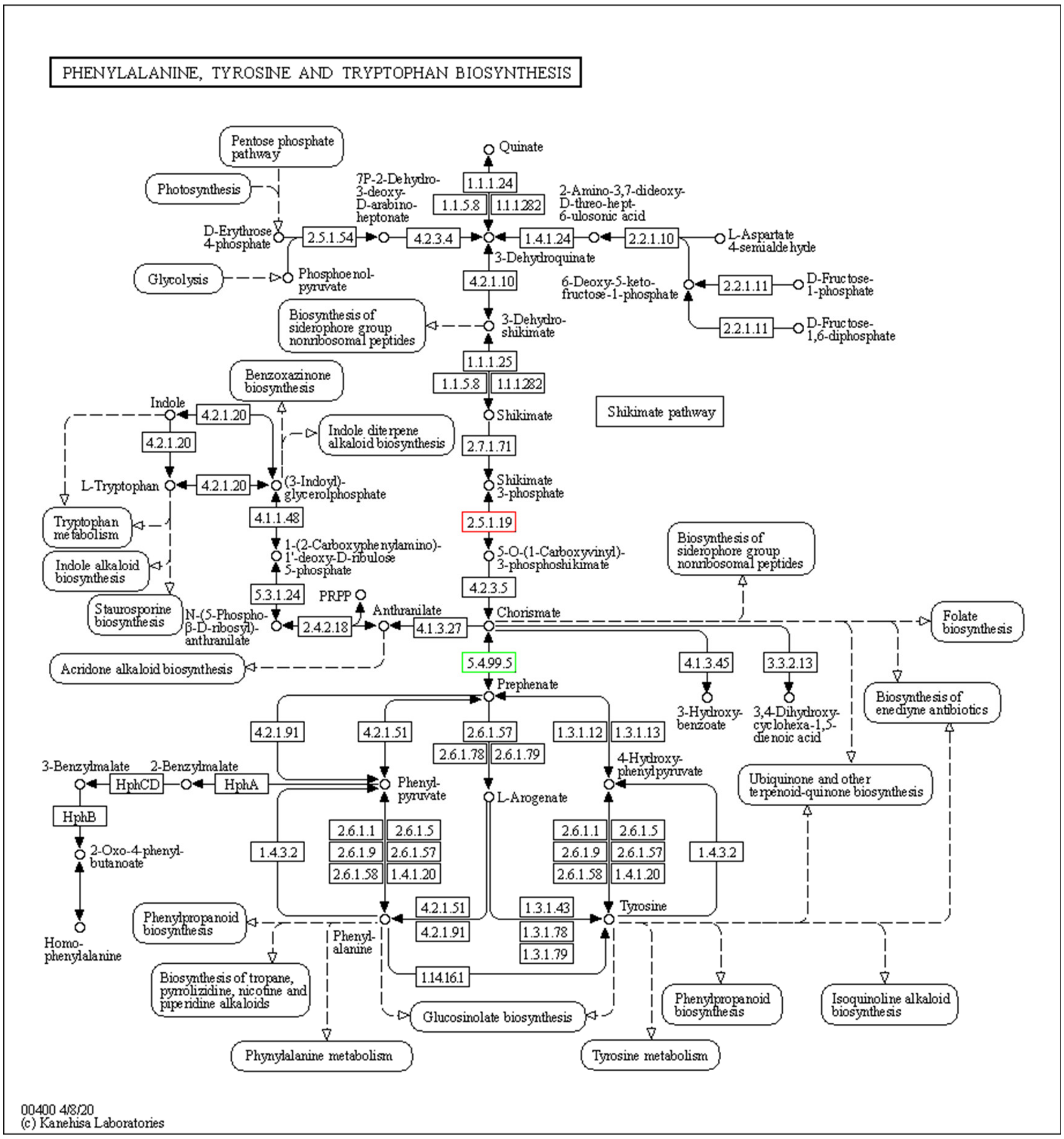

Figure S1. DEPs of BBL/ZH58 enriched in protein processing in the endoplasmic reticulum. Red indicates relatively high expression, green indicates relatively low expression, and white indicates the same expression levels in the two lines.

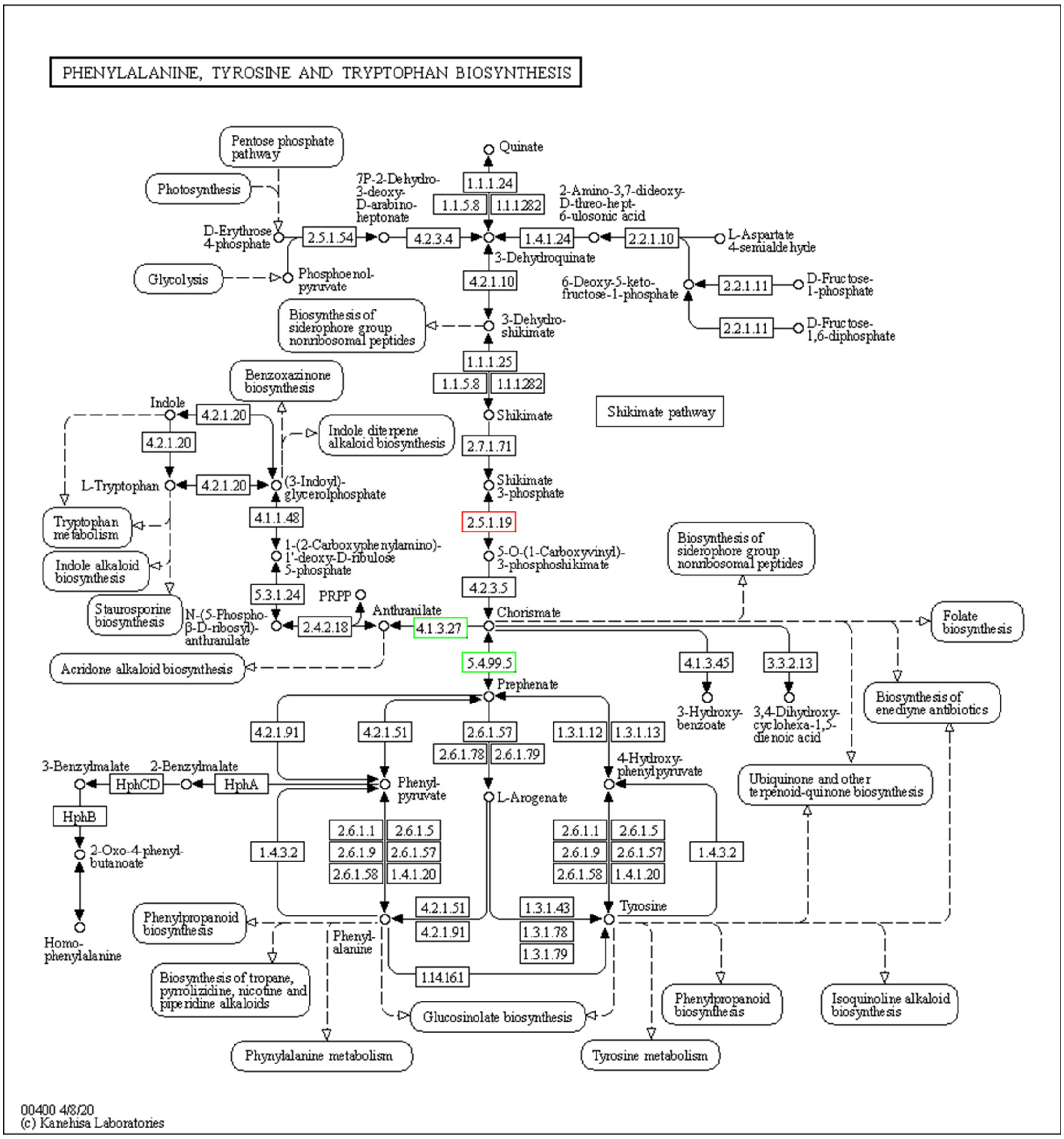

Figure S2. DEPs of BFL-1/ZH58 enriched in protein processing in the endoplasmic reticulum. Red indicates relatively high expression, green indicates relatively low expression, and white indicates the same expression levels in the two lines.

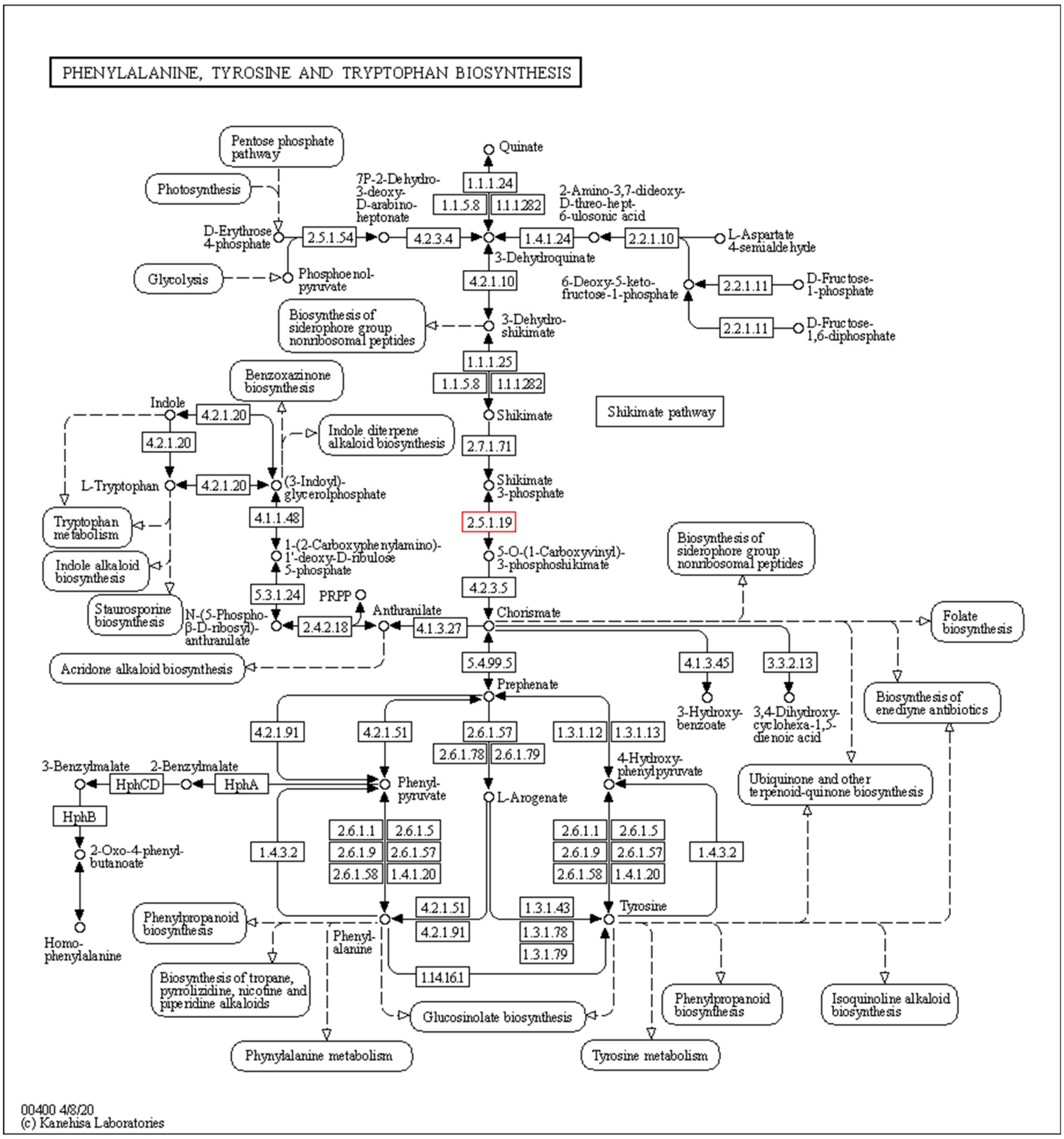

Figure S3. DEPs of BFL-2/ZH58×CH72 enriched in protein processing in the endoplasmic reticulum. Red indicates relatively high expression, green indicates relatively low expression, and white indicates the same expression levels in the two lines.

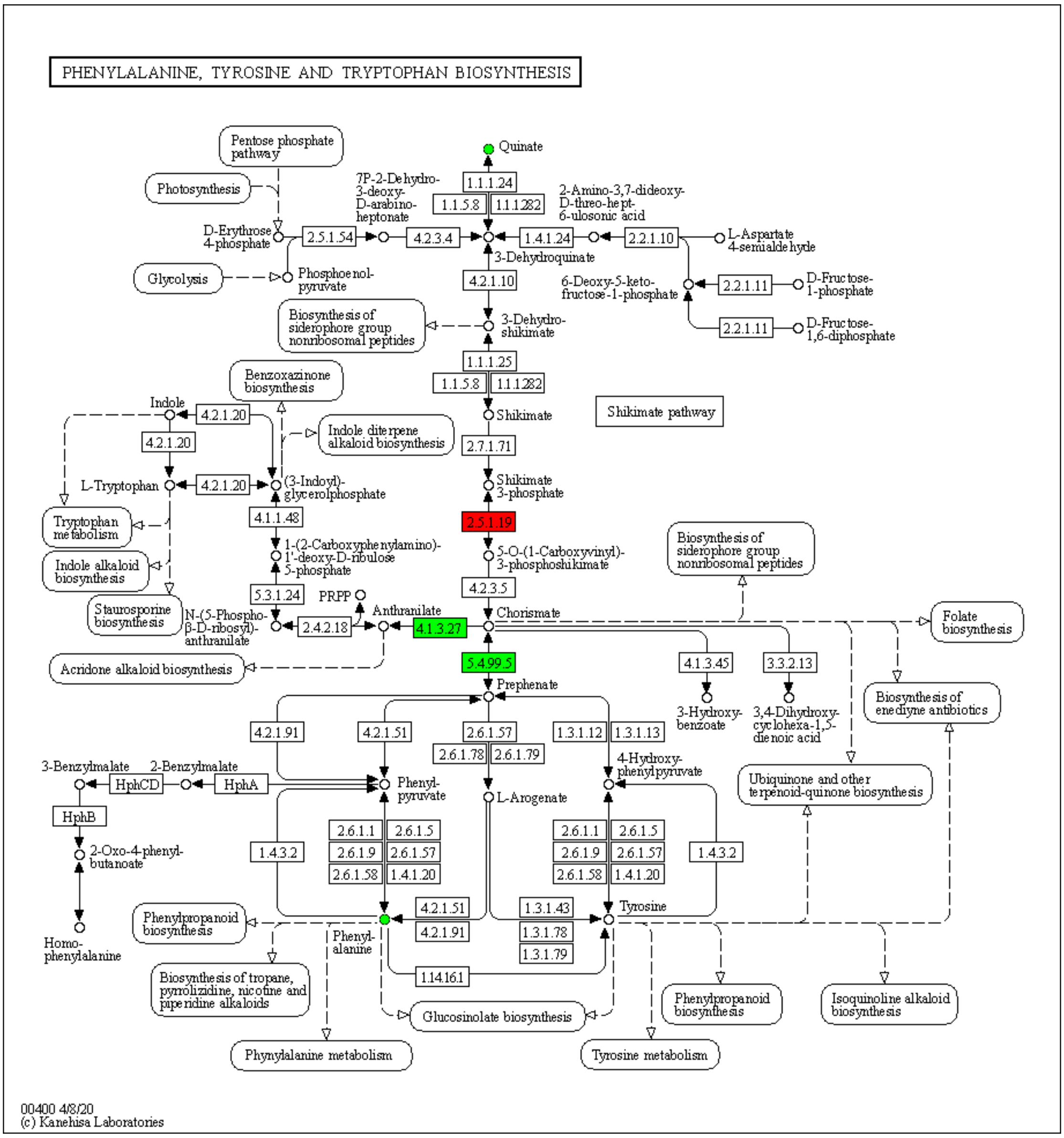

Figure S4. The integrated analyses of DEPs and DAMs of BFL-1/ZH58 enriched in protein processing in the endoplasmic reticulum. Red indicates relatively high expression, green indicates relatively low expression, and white indicates the same expression levels in the two lines. Square indicates protein, and circle indicates metabolite.

Table S1. Primers for PCR detection of transgenic maize.

Primers	Sequence of primers（5'-3'）	Size of fragment（bp）
BBL2-2 event-specific F	TACTAGATCGGGAATTAAACTATCAGTG	168
BBL2-2 event-specific R	ACCTGGCACAGCACACATAGAG	
BFL4-2 event-specific F	GGCAATAAGAGTACAAAGATGAACA	271
BFL4-2 event-specific R	AGATCGGGAATTAAACTATCAGTGT	
BBL2-2 EPSPS F	CGGTCCTCCACGCTGAAG	444
BBL2-2 EPSPS R	AGCCTTCGTATCGGAGAGT	
BFL4-2 EPSPS F	CATTCCAGGCGACAAGTCTATCT	662
BFL4-2 EPSPS R	CGCCTGGGACATCAATCACT	
Cry1Ab F	ATGGACAACAACCCGAACA	829
Cry1Ab R	CGAAGTTCTCCAGGACGGG	
Cry3Bb F	AGTCGTTCCTCAACACCATCTG	593
Cry3Bb R	GCGGATGTCGTAGAAGGGG	
Cry1F F	ACAACATCCAGAACCAGTGCG	1951
Cry1F-R	CAGAACTCGTCAGAGAGGCACT	

Table S2. The list of DEPs in BBL/ZH58.

Accession	Description	Mol. weight [kDa]	BBL/ZH58	BBL/ZH58 PVal	BBL/ZH58 State
epsps	epsps	47.614	172.77	1.46348×10^{-6}	up
Cry1Ab	Cry1Ab	91.956	3.199	0.000658169	up
O64964	Aquaporin TIP1-1	25.821	0.464	3.98072×10^{-7}	down
Q94IM1	22 kDa alpha-zein 14	28.934	5.72	3.50994×10^{-13}	up
P04706	Glutelin-2	23.688	0.351	4.03766×10^{-8}	down
P06673	Zein-beta	19.531	2.088	8.37627×10^{-10}	up
P49106	14-3-3-like protein GF14-6	29.662	0.422	2.42053×10^{-5}	down
P81008	Defensin-like protein 1	5.199	2.152	1.53938×10^{-7}	up
P83506	Probable non-specific lipid-transfer protein 2	7.3115	2.375	5.92951×10^{-5}	up
Q41819	Indole-3-acetate beta-glucosyltransferase	49.71	2.077	4.1027×10^{-7}	up
A0A1D6DXF6	Eukaryotic translation initiation factor 3 subunit C	106.06	2.012	1.19981×10^{-9}	up
A0A1D6EWX3	Aconitate hydratase	107.26	0.105	4.9225×10^{-8}	down
A0A1D6F072	Indole-3-acetic acid amido synthetase	67.209	0.375	2.36457×10^{-5}	down
A0A1D6GGD7	Allene oxide synthase chloroplastic	56.583	2.2	1.9631×10^{-5}	up
A0A1D6HBQ7	Uncharacterized protein	15.344	0.411	3.66757×10^{-5}	down
A0A1D6HBT3	Glycine-rich cell wall structural protein	11.918	2.248	8.01337×10^{-7}	up
A0A1D6HEN7	Vacuolar processing enzyme 1	54.623	0.413	1.09034×10^{-9}	down
A0A1D6HUR0	Uncharacterized protein	19.01	2.882	0.000147939	up
A0A1D6I4V5	Alanine aminotransferase 2 mitochondrial	50.682	4.425	2.59007×10^{-11}	up
A0A1D6IN22	Glycosyltransferase	52.397	2.408	6.13933×10^{-11}	up
A0A1D6JTL4	Caleosin	27.243	0.069	1.07129×10^{-7}	down
A0A1D6KSA0	Tonoplast intrinsic protein3	27.361	2.071	2.78847×10^{-5}	up
B4F817	Dehydroascorbate reductase	23.419	2.013	1.26182×10^{-7}	up
A0A1D6M3X4	Peptidase A1 domain-containing protein	44.884	2.29	5.90069×10^{-10}	up
A0A1D6MA73	Pistil-specific extensin-like protein	21.086	2.365	1.5193×10^{-7}	up
A0A1D6N932	Osmotin-like proteinM34	23.069	2.141	4.55393×10^{-10}	up
A0A804LG03	Uncharacterized protein	58.155	0.413	3.53154×10^{-14}	down
A0A804LUU9	Uncharacterized protein	22.364	2.426	3.32839×10^{-8}	up
A0A804MGL2	Uncharacterized protein	14.571	0.24	1.56129×10^{-9}	down
A0A804MXV9	Uncharacterized protein	40.208	18.256	0.000108459	up

A0A804N5U7	Uncharacterized protein	39.666	0.482	1.95009×10^{-7}	down
A0A804NWX6	Uncharacterized protein	28.88	3.396	3.09982×10^{-6}	up
A0A804P0S1	Uncharacterized protein	41.341	0.148	9.0846×10^{-11}	down
A0A804P6E1	Uncharacterized protein	13.366	2.275	7.75334×10^{-7}	up
A0A804PEW7	Uncharacterized protein	44.627	4.918	0.009749018	up
A0A804PRB2	Uncharacterized protein	34.268	4.239	1.27434×10^{-9}	up
A0A804Q0J2	Uncharacterized protein	27.573	2.109	6.30083×10^{-6}	up
B6TFF1	Thioredoxin O1 mitochondrial	19.265	0.477	3.55645×10^{-6}	down
A0A804UML8	Uncharacterized protein	59.746	0.291	9.65224×10^{-7}	down
B4F922	Acyltransferase	47.58	0.376	7.1074×10^{-6}	down
B4F9D6	Hevein-like preproprotein	15.716	5.414	0.000340742	up
B4FB66	40S ribosomal protein S5-2	22.196	0.481	3.02683×10^{-6}	down
B4FFB8	5a2 protein	12.046	0.392	5.07644×10^{-9}	down
B4FFQ0	Thioredoxin	13.608	0.349	9.30389×10^{-6}	down
B4FHT1	Annexin	35.344	0.304	3.15073×10^{-11}	down
B4FI88	60S acidic ribosomal protein P2-5	11.329	0.418	1.28648×10^{-8}	down
B4FKP4	Peroxygenase 2	34.23	0.305	8.0305×10^{-10}	down
B4FN23	Dirigent protein	34.528	3.024	8.43224×10^{-12}	up
B4FPE4	V-type proton ATPase subunit G	12.235	2.005	0.000361481	up
B4FQ44	L-tryptophan--pyruvate aminotransferase 1	47.217	0.467	3.28863×10^{-8}	down
B4FR99	Acidic endochitinase	32.535	12.451	2.47878×10^{-12}	up
B4FRA6	OSJNBb0091E11.19-like protein	40.523	3.194	1.97919×10^{-10}	up
B4FRP8	Plastid-lipid-associated protein 2	34.28	2.643	3.65334×10^{-6}	up
B4FSD4	Acetylglutamate kinase chloroplastic	35.422	2.132	9.37342×10^{-7}	up
B4FVP5	Pathogenesis-related protein4	17.224	2.352	3.4396×10^{-5}	up
B4G0F8	PRA1 family protein	24.271	2.019	0.00020015	up
B4G1C2	GH18 domain-containing protein	34.236	2.354	1.76815×10^{-10}	up
B4G1T3	Chitinase chem5	30.293	6.324	0.001093716	up
B6SGF3	Glyoxalase family protein superfamily	15.083	0.058	7.23914×10^{-16}	down
B6SH12	Win1	16.092	2.717	1.01676×10^{-6}	up
B6SID7	Late embryogenesis-abundant protein, group 3	18.599	2.002	5.27554×10^{-12}	up
B6SII5	Uncharacterized protein	25.343	2.904	3.20539×10^{-6}	up
B6SN63	Late embryogenesis-abundant protein 3	21.306	2.101	3.9408×10^{-9}	up
B6SZ78	Cytochrome P450 CYP71W7	57.855	2.584	0.003784056	up

B6T391	Lichenase-2	34.973	0.439	4.32464×10^{-8}	down
B6TF54	Chorismate mutase	35.005	0.31	1.26995×10^{-8}	down
B6UH04	Subtilisin-chymo-trypsin inhibitor CI-1B	7.9792	6.17	1.89611×10^{-11}	up
B6UH67	Late embryogenesis-abundant protein D-34	27.177	2.162	1.18383×10^{-9}	up
B7ZZ42	Heat shock 70 kDa protein 3	71.162	0.38	4.79722×10^{-10}	down
C0HGH7	Universal stress family protein	21.162	2.183	0.001865446	up
C0P8L5	Uncharacterized protein	27.093	0.138	1.515×10^{-8}	down
C0PAS9	Alba DNA/RNA-binding protein	15.873	2.255	7.47414×10^{-5}	up
C0PIH6	Aldose 1-epimerase	40.62	0.398	1.70202×10^{-9}	down
C4JBA7	Uncharacterized protein	24.661	0.439	6.53505×10^{-5}	down
K7VQ98	Class I heat shock protein 3	15.846	2.002	3.45108×10^{-7}	up
Q8W0V2	Lipoxygenase	96.489	3.412	1.26592×10^{-12}	up

Table S3. The list of DEPs in BFL-1/ZH58.

Accession	Description	Mol. weight [kDa]	BFL-1/ZH58	BFL-1/ZH58 PVal	BFL-1/ZH58 State
epsps	epsps	47.614	23.718	0.000121842	up
Q94IM1	22 kDa alpha-zein 14	28.934	3.913	8.87292×10^{-10}	up
P09233	Superoxide dismutase [Mn] 3.1, mitochondrial	25.545	2.115	0.000179006	up
Q6XZ78	Fructokinase-2	35.481	0.442	1.9337×10^{-9}	down
Q9FQA3	Glutathione transferase GST 23	24.878	2.075	3.42691×10^{-9}	up
A0A1D6DXF6	Eukaryotic translation initiation factor 3 subunit C	106.06	2.105	6.242×10^{-9}	up
A0A1D6F072	Indole-3-acetic acid amido synthetase	67.209	0.245	4.80815×10^{-7}	down
A0A1D6HTN8	Xylanase inhibitor protein 1	32.992	2.589	6.57885×10^{-8}	up
A0A1D6HUR0	Uncharacterized protein	19.01	2.679	0.001651847	up
A0A1D6I4V5	Alanine aminotransferase 2 mitochondrial	50.682	2.31	4.74785×10^{-9}	up
A0A1D6IKD3	Uncharacterized protein	37.52	2.106	6.02537×10^{-8}	up
B6T7W2	Copper transport protein family	12.726	0.418	1.60392×10^{-6}	down
A0A1D6LA97	Anthranilate synthase	64.487	0.475	8.69049×10^{-7}	down
A0A1D6N0K3	Peroxidase	38.089	0.311	0.002788432	down
A0A1D6N323	Stearoyl-acyl-carrier-protein desaturase1	42.74	0.397	1.94238×10^{-6}	down
A0A1D6NFL0	Glutamate synthase (NADH)	236.82	0.459	1.505×10^{-7}	down
A0A804LSV9	Uncharacterized protein	33.111	2.194	5.12458×10^{-5}	up
A0A804M4S4	Uncharacterized protein	12.923	2.687	0.02095343	up

A0A804MXV9	Uncharacterized protein	40.208	5.141	0.001977593	up
A0A804NKN1	Uncharacterized protein	13.984	2.045	8.66456×10^{-9}	up
A0A804NLI1	Uncharacterized protein	52.448	0.417	3.10403×10^{-7}	down
A0A804QW48	Uncharacterized protein	51.653	0.425	0.01349713	down
A0A804UML8	Uncharacterized protein	59.746	0.409	1.99729×10^{-7}	down
B4F922	Acyltransferase	47.58	0.366	2.08369×10^{-8}	down
B4FFQ0	Thioredoxin	13.608	0.345	3.89812×10^{-9}	down
B4FNY2	Translin family protein	32.261	0.425	0.004111655	down
B4FR99	Acidic endochitinase	32.535	7.692	1.35695×10^{-11}	up
B4FRA6	OSJNBb0091E11.19-like protein	40.523	2.357	1.90028×10^{-9}	up
B4FRP8	Plastid-lipid-associated protein 2	34.28	2.14	1.49975×10^{-5}	up
B4FSR6	Glutathione S-transferase	26.491	2.03	0.003197439	up
B4G197	16.9 kDa class I heat shock protein 1	17.223	2.402	2.24475×10^{-7}	up
B6SMQ5	Triose phosphate isomerase5	27.339	0.489	4.5812×10^{-5}	down
B6T391	Lichenase-2	34.973	0.475	1.06673×10^{-7}	down
B6TF54	Chorismate mutase	35.005	0.39	9.07098×10^{-9}	down
B7ZZ42	Heat shock 70 kDa protein 3	71.162	0.35	5.68537×10^{-12}	down
C0P8L5	Uncharacterized protein	27.093	0.471	7.49139×10^{-6}	down
C0PGB5	Pyruvate kinase	62.638	0.449	1.49176×10^{-6}	down
E1AFV5	Beta-1,3-glucanase	35.976	2.132	4.4876×10^{-10}	up
K7VQ65	Putative translation elongation factor family protein	93.906	0.49	5.56232×10^{-7}	down
Q8W0V2	Lipoxygenase	96.489	2.04	1.02282×10^{-10}	up

Table S4. The list of DEPs in BFL-2/ZH58×CH72.

Accession	Description	Mol. weight [kDa]	BFL-2/ZH58×CH72	BFL-2/ZH58×CH72 PVal	BFL-2/ZH58×CH72 State
epsps	epsps	47.614	16.396	1.3585×10^{-7}	up
Cry1F	Cry1F	73.876	26.586	0.004223503	up
A5H8G4	Peroxidase 1	38.354	0.39	3.50531×10^{-5}	down
O24581	Luminal-binding protein 3	73.156	0.404	0.001158105	down
P08031	16 kDa gamma-zein	19.558	2.036	0.000261263	up
P11155	Pyruvate, phosphate dikinase 1, chloroplastic	102.67	2.707	3.07029×10^{-5}	up
P24632	17.8 kDa class II heat shock protein	17.799	2.34	9.58835×10^{-10}	up
Q41819	Indole-3-acetate beta-glucosyltransferase	49.71	2.151	9.12371×10^{-7}	up
A0A1D6HTN8	Xylanase inhibitor protein 1	32.992	3.619	3.14646×10^{-10}	up

A0A1D6L429	Late embryogenesis-abundant protein 31	21.194	0.399	9.64566×10^{-6}	down
A0A1D6M439	Pyruvate, phosphate dikinase	83.419	2.597	8.36256×10^{-10}	up
A0A804MXV9	Uncharacterized protein	40.208	4.111	0.022128596	up
A0A804QA55	Uncharacterized protein	43.647	0.402	6.33373×10^{-8}	down
B4F976	17.4 kDa class I heat shock protein	17.88	2.713	9.0913×10^{-6}	up
B4F9D6	Hevein-like preproprotein	15.716	2.669	1.64697×10^{-10}	up
B4FR99	Acidic endochitinase	32.535	2.592	9.81667×10^{-8}	up
B4FUN3	GST N-terminal domain-containing protein	27.324	3.458	2.68607×10^{-5}	up
B4FVP5	Pathogenesis-related protein4	17.224	2.291	1.20073×10^{-6}	up
B4G0F8	PRA1 family protein	24.271	2.108	2.1691×10^{-5}	up
B4G1T3	Chitinase chem5	30.293	3.906	5.61178×10^{-7}	up
B6T522	40S ribosomal protein S14	16.359	2.109	0.019758588	up
C0PAJ0	ADP-ribosylation factor A1F	20.627	2.147	0.000263885	up
C4JBA7	Uncharacterized protein	24.661	0.485	0.000770984	down
K7UCZ5	Heat shock 70 kDa protein 6 chloroplastic	74.67	2.016	0.000350693	up
K7WCU4	Uncharacterized protein	14.081	0.498	3.03405×10^{-6}	down

Table S5. The list of DAMs in ZH58/BBL.

Index	Compounds	VIP	Fold_Change	Type
pmp000235	Salcolin B*	1.36	2.12×10^{-1}	down
pmb0501	Agmatine	1.34	3.05	up
mws4085	Sinapic acid	1.29	4.48×10^{-1}	down
Lmyn004037	Eriodictyol-7-O-(6''-O-galloyl)glucoside	1.34	4.79	up
mws0983	N-Oleoylethanolamine	1.37	2.59	up
Hmbp006861	1,2-O-Diferuloylglycerol*	1.36	4.60	up
pmp000091	1,3-O-Diferuloylglycerol*	1.36	4.60	up
pme1014	Menatetrenone (Vitamin K2)	1.39	1.72×10^{4}	up
HJN092	p-Coumaroylferuloylputrescine	1.36	2.54	up
MWSmce083	Ferulic acid methyl ester	1.26	4.89×10^{-1}	down
mws0918	Prunetin (5,4'-Dihydroxy-7-methoxyisoflavone)	1.03	3.65×10^{-1}	down
pmn001690	3-Hydroxy-4-isopropylbenzylalcohol-3-O-glucoside	1.32	4.05	up
Lmhp009190	2-Linoleoylglycerol-1,3-di-O-glucoside*	1.33	2.29	up
Zmdp000292	Arginine methyl ester	1.33	2.12	up
Lmbn004685	5S,8R-DiHODE; (5S,8R,9Z,12Z)-5,8-Dihydroxyoctadeca-9,12-dienoate	1.14	4.01×10^{-1}	down
Lmbn007891	Hydroxy ricinoleic acid	1.39	2.63	up
pmb3042	Tricin-5-O-Glucoside	1.36	4.70×10^{-1}	down
pme1109	Guanine*	1.31	2.17	up
Lmlp001118	3'-Glucosyl-6,7-dihydroxy-N-methyl-benzyltetrahydroisoquinoline	1.26	2.37	up
Lmyn006011	Gingerglycolipid B*	1.37	2.22	up
mws0126	LysoPC 18:0*	1.39	2.01	up
Hmqp006023	Ethyl 9-Hydroxy-10,12-octadecadienoic acid	1.36	2.61	up

Hmcp002187	Limocitrin-3-O-galactoside	1.06	4.21×10^{-1}	down
pme2914	3-Hydroxy-3-methylpentane-1,5-dioic acid	1.31	4.70×10^{-1}	down
pmb3142	Salicylic acid-2-O-glucoside	1.30	4.62	up
pme1841	Cadaverine	1.22	4.40×10^{-1}	down
MWSmce415	Cirsimaritin (4',5-dihydroxy-6,7-dimethoxyflavone)*	1.34	2.30×10^{-1}	down
Hmcn003273	Reynosin	1.36	3.30×10^{-1}	down
Xmyn008071	Gnetifolin B	1.29	4.14×10^{-1}	down
pme2776	2'-Deoxyinosine	1.37	2.07×10^{-1}	down
pme0021	L-Phenylalanine	1.33	2.39	up
Lmlp003161	N-Feruloylputrescine	1.38	9.21	up
pmn001694	9,10,13-Trihydroxy-11-Octadecenoic Acid	1.38	2.17	up
pme1178	Guanosine	1.30	2.20	up
pmn001689	9-Hydroxy-12-oxo-15(Z)-octadecenoic acid	1.39	2.53	up
pmp000234	Salcolin A*	1.38	2.49×10^{-1}	down
pme3011	γ-Aminobutyric acid	1.31	4.30×10^{-1}	down
pmb0746	Tricin-4'-O-(guaiacylglycerol)ether	1.39	2.06×10^{-1}	down
pme3967	2-(Dimethylamino)guanosine	1.33	4.94	up
Zmjp000182	N-Monomethyl-L-arginine	1.34	2.21	up
pme0026	L-Lysine	1.32	7.02	up
pmb0296	1-Oleoyl-Sn-Glycerol	1.38	2.62	up
Hmhp005160	Rehderianin I	1.39	6.73×10^{-2}	down
Lmyn005812	Gingerglycolipid A*	1.36	2.01	up
pmn001319	1-O-Feruloyl-3-O-p-Coumaroylglycerol	1.34	4.82	up
Lmyn006221	Gingerglycolipid C	1.38	2.59	up
pme3337	Succinyladenosine	1.35	3.08	up
Jmzn006005	3,4-Methylenedioxy cinnamyl alcohol	1.14	4.72×10^{-1}	down
Zmyn000155	N-α-Acetyl-L-ornithine	1.31	2.31	up
Hmxp007521	3',4'-Dihydroxy-7,5'-dimethoxyflavone*	1.37	2.29×10^{-1}	down
Hmqn003054	9,10,11-Trihydroxy-12-octadecenoic acid	1.37	2.22	up
mws1200	Trans-4-Hydroxycinnamic Acid Methyl Ester	1.15	3.44×10^{-1}	down
Lmbn004240	9,10-Dihydroxy-12,13-epoxyoctadecanoic acid	1.37	2.02	up
MWSmce548	Betaine	1.34	3.66×10^{-1}	down
Lmhp009384	1-Linoleoylglycerol-2,3-di-O-glucoside*	1.38	2.20	up
Qmqp101713	Loliolide*	1.22	2.34	up
pme3033	N,N-Dimethylglycine	1.34	4.67×10^{-1}	down
Lmhp009890	LysoPC 20:3	1.38	5.00	up
MWS1882	Iminodiacetic acid	1.34	2.91	up
Lmcp002302	N6-(2-Hydroxyethyl)adenosine	1.33	5.32	up
pmb0745	Tricin-4'-O-syringyl alcohol	1.35	1.74×10^{-1}	down
pmc0274	6-Methylmercaptopurine	1.31	2.51	up
Zmzp006857	Tricin-4'-O-[β-guaiacyl-(9''-O-acetyl)glycerol]ether	1.37	9.29×10^{-2}	down
pme3961	2'-Deoxyadenosine	1.37	2.93×10^{-1}	down
Zmhp004065	7,8-Dihydroxy-5,6,4'-trimethoxyflavone*	1.27	2.11×10^{-1}	down
MWSmce609	L-Aspartic acid	1.34	2.20	up
pme0243	Glutaric acid	1.38	1.94×10^{-1}	down
mws0639	2,3-Dihydroxybenzoic Acid*	1.36	4.95×10^{-1}	down
MWSmce295	1-beta-D-Arabinofuranosyluracil	1.34	2.20	up
pmb0716	Tricin-4'-O-glucoside-7-O-glucoside*	1.33	2.56×10^{-1}	down
Lmjp004941	3,5,4'-Trihydroxy-7-methoxyflavone (Rhamnocitrin)	1.29	4.20×10^{-1}	down
mws1401	L-Theanine	1.33	3.11×10^{-1}	down
Zmhn001446	Syringaresinol-4'-O-glucoside	1.23	8.86×10	up
pmn001610	Eicosadienoic acid	1.00	3.29	up
Jmcp010061	Costic acid	1.35	3.32×10^{-1}	down
Zmhn002301	p-Coumaric acid-4-O-glucoside	1.28	3.02	up
pme3388	Homoarginine	1.35	2.23	up
Lmgn000160	3-Ureidopropionic Acid	1.28	2.17	up
mws0001	L-Asparagine	1.29	2.12	up
mws0133	Nicotinamide	1.38	2.95	up

Zmhp003514	6,7,8-Tetrahydroxy-5-methoxyflavone	1.24	2.81×10^{-1}	down
MWSslk174	2,4'-Dihydroxybenzophenone	1.33	3.29×10^{-1}	down
pme0193	L-Glutamine	1.35	4.61	up
pmb0962	L-Lysine-Butanoic Acid	1.34	1.50×10^{-1}	down
MWSHY0069	Hispidulin (5,7,4'-Trihydroxy-6-methoxyflavone)	1.33	3.33×10^{-1}	down
Lmyp001747	2-O-Glucosyl-7-hydroxy-1,4(2H)-benzoxazin-3-one (DHBOA glucoside)	1.14	2.37	up
pmb0490	p-Coumaroylputrescine	1.39	6.77	up
MWSslk060	Eupatilin (5,7-Dihydroxy-3',4',6-Trimethoxyflavone)	1.31	2.30×10^{-1}	down
Lmsn009824	2α,3β,19α,23-Tetrahydroxyolean-12-en-28-oic acid	1.38	2.36×10^{-1}	down
Hmhp005846	Tenaxin I	1.33	1.99×10^{-1}	down
Zmxp004503	Tricin-5,7-O-diglucoside*	1.14	4.78×10^{-1}	down
mws0183	3,4-Dihydroxybenzoic acid (Protocatechuic acid)*	1.36	4.71×10^{-1}	down
MWSmce577	(E)-Ethyl p-methoxycinnamate	1.39	1.56×10^{3}	up
Lmhp008513	2-α-Linolenoyl-glycerol-1,3-di-O-glucoside*	1.22	2.18	up
Zmyn005209	12-Methyltetradecanoic Acid*	1.39	4.16×10^{-5}	down
mws0182	4-Hydroxyphenylacetic acid	1.21	2.30	up
mws0129	Genkwanin (Apigenin 7-methyl ether)	1.05	3.80×10^{-1}	down
mws0473	2-Methylsuccinic acid	1.36	1.84×10^{-1}	down
Lmmn000806	Dimethylmalonic acid	1.36	1.84×10^{-1}	down
Lmmn002164	Monomethyl succinate	1.36	1.84×10^{-1}	down
Jmcp009989	Pterodontic acid	1.36	3.26×10^{-1}	down
Lmzp004885	Tricin (5,7,4'-Trihydroxy-3',5'-dimethoxyflavone)	1.39	7.14×10^{-2}	down
Lmgp004539	5,7,4'-Trihydroxy-6,8-dimethoxyisoflavone-7-O-galactoside-glucoside	1.29	4.50×10^{-1}	down
pme3034	Ethylmalonic acid	1.37	2.05×10^{-1}	down
pme1173	Allopurinol	1.33	3.04	up
Smpn009074	2α,3α,19α,23-tetrahydroxy-12-ursen-28-oic acid	1.39	4.90×10^{-4}	down
zjzp121501	Eupatorinol	1.34	3.51×10^{-1}	down
pmb3053	Tricin-4'-O-eudesmic acid	1.38	1.59×10^{-1}	down
Zmjp004852	5,6,3',4'-Tetrahydroxy-3,7-dimethoxyflavone-6-O-glucoside	1.24	2.56×10^{-1}	down
Hmtp000776	4,5,6-Trihydroxy-2-cyclohexen-1-ylideneacetonitrile	1.23	2.02	up
pmb2795	4-Methoxycinnamic acid	1.17	3.32×10^{-1}	down
pme2527	L-Ornithine	1.33	2.02	up
mws1195	p-Coumaric acid methyl ester	1.39	4.07×10^{-4}	down
mws1038	Pantetheine	1.20	2.01	up
pme2693	N-Acetylputrescine	1.21	3.11	up
Hmbn002692	6'-O-Feruloyl-D-sucrose	1.30	3.99×10^{-1}	down
Lmhp008744	1-α-Linolenoyl-glycerol-2,3-di-O-glucoside*	1.30	2.75	up
MWSmce613	Citicoline	1.22	2.07	up
MWS5209	N-Methyl-α-aminoisobutyric acid	1.29	1.56×10^{-1}	down
pmb0496	N-Feruloylagmatine	1.38	4.88	up
Wmhp000055	2,5-Dimethoxybenzoquinone	1.26	2.55	up
mws0520	N-Acetyl-L-tyrosine	1.26	2.58×10^{-1}	down
MWSHY0009	Diosmetin (5,7,3'-Trihydroxy-4'-methoxyflavone)	1.24	3.63×10^{-1}	down
Zmdp001647	γ-Glutamyl-L-valine	1.15	2.16	up
pma0633	N-p-Coumaroylspermine	1.29	4.10×10^{-1}	down
mws0248	Uridine	1.12	2.41	up
Zmzp000145	Trimethyllysine	1.17	2.62	up
MWSmce498	Apigenin-7-O-neohesperidoside (Rhoifolin)*	1.19	2.86	up
Zmsp000366	Fagomine	1.12	2.03	up
mws0005	Tryptamine	1.34	3.55	up
pmb2857	L-Glutamic acid-O-glycoside	1.05	2.07	up
Lmmn002179	Methyl salicylate-2-O-glucoside	1.35	1.51×10	up
Lmmp002013	Dihydroferuloylputrescine	1.38	1.11×10	up
pmp000548	Pratensein	1.27	3.66×10^{-1}	down
HJAP035	1-(4-Methoxyphenyl)allyl acetate	1.31	3.00	up
pmb2620	3,4-Dimethoxycinnamic acid	1.13	4.79×10^{-1}	down

Lmlp002205	Isololiolide*	1.18	2.22	up
mws1060	9-(Arabinosyl)hypoxanthine	1.29	3.30	up
Zmhn003257	5,7,2'-Trhiyroxy-8-methoxyflavone	1.20	4.54×10^{-1}	down
pme0368	Apigenin-7-O-rutinoside (Isorhoifolin)*	1.33	2.36	up
pmn001419	1-O-p-Coumaroyl-β-D-glucose	1.27	2.70	up
mws0856	Quercetin-4'-O-glucoside (Spiraeoside)	1.03	4.45×10	up
pmb3031	Tricin-4'-O-glycerol	1.16	6.14×10^{-2}	down
Lmpp003930	Apigenin-7-O-(6''-p-Coumaryl)glucoside	1.16	2.03	up
Hmgp002564	Tricin-7-O-glucuronyl(2→1)glucuronide	1.37	2.48×10^{-1}	down

Table S6. The list of DAMs in ZH58/BFL-1.

Index	Compounds	VIP	Fold_Change	Type
Lmhp112042	1-Linoleoylglycerol*	1.38	2.43	up
Lmyn002540	Dioxindole-3-acetyl-3-O-glucoside	1.38	2.50	up
pmb0501	Agmatine	1.37	5.09	up
Zmyn004732	2R-hydroxy-9Z,12Z,15Z-octadecatrienoic acid	1.39	2.16	up
mws0275	L-Malic acid	1.39	4.74	up
Zmdp000376	4-Guanidinobutanal	1.33	2.93	up
Lmyn004037	Eriodictyol-7-O-(6''-O-galloyl)glucoside	1.35	2.62	up
mws0983	N-Oleoylethanolamine	1.37	2.95	up
Zmyn004449	9-Hydroxy-12-oxo-10(E),15(Z)-octadecadienoic acid	1.39	2.34	up
mws0192	Succinic acid	1.33	2.15	up
Lmbn000198	3-Dehydro-L-Threonic Acid	1.37	4.18	up
mws0281	Citric Acid*	1.38	3.95	up
Lmhp011388	2-α-Linolenoyl-glycerol*	1.39	3.09	up
mws0216	Trans-4-Hydroxy-L-proline*	1.36	2.18	up
pmf0348	2,6-Dimethyl-7-octene-2,3,6-triol	1.38	2.28	up
Lmhp008833	LysoPC 16:1(2n isomer)*	1.05	2.95	up
mws0277	Quinic Acid	1.37	2.38	up
pmn001320	1-O-p-Cumaroylglycerol	1.35	2.36	up
Lmbn003524	9-Oxononanoic acid	1.30	2.01	up
Zmyn004676	17-Hydroxylinolenic acid	1.33	2.61	up
Ymjm000140	3-O-Caffeoylquinic acid methyl ester	1.26	2.64	up
Lmhp009190	2-Linoleoylglycerol-1,3-di-O-glucoside*	1.32	2.50	up
Zmpn003368	13S-Hydroxy-9Z,11E,15Z-octadecatrienoic acid	1.38	2.20	up
Zmgn000503	2,3-Dihydroxy-3-Methylbutanoic Acid	1.36	3.12	up
pmb2787	9-Oxo-10E,12Z-octadecadienoic acid	1.38	2.14	up
pme1474	5'-Deoxy-5'-(methylthio)adenosine	1.34	3.93	up
mws0237	Azelaic acid	1.38	2.33	up
Lmhp011562	1-α-Linolenoyl-glycerol*	1.39	2.94	up
Lmbn007891	Hydroxy ricinoleic acid	1.39	3.32	up
pme0183	Isoguanine*	1.35	2.34	up
pme1109	Guanine*	1.31	2.79	up
Lmhp005784	p-Coumaroylferuloylcadaverine	1.36	3.75×10^{-1}	down
Lmyn006011	Gingerglycolipid B*	1.39	2.87	up
Hmqp006023	Ethyl 9-Hydroxy-10,12-octadecadienoic acid	1.37	3.03	up
pme2914	3-Hydroxy-3-methylpentane-1,5-dioic acid	1.20	9.73	up
pmb3142	Salicylic acid-2-O-glucoside	1.32	2.07	up
MWSmce415	Cirsimaritin (4',5-dihydroxy-6,7-dimethoxyflavone)*	1.19	2.02	up
Hmcn003273	Reynosin	1.34	2.33	up
mws0147	β-Hydroxyisovaleric acid	1.26	2.48	up
pme0274	6-Aminocaproic acid	1.32	2.48	up
pme0021	L-Phenylalanine	1.36	3.44	up
Lmlp003161	N-Feruloylputrescine	1.38	1.31×10	up
pmn001694	9,10,13-Trihydroxy-11-Octadecenoic Acid	1.39	2.23	up
pmp001287	N-Benzylmethylene isomethylamine	1.29	2.77	up
pme1178	Guanosine	1.35	3.47	up
Zmtn001624	N-Acetylisatin	1.37	3.17	up

pmb2228	LysoPC 19:0	1.37	2.26	up
pmn001689	9-Hydroxy-12-oxo-15(Z)-octadecenoic acid	1.39	4.07	up
pmn001658	Cinnzeylanine	1.38	3.66	up
HJN003	1-O-Sinapoyl-β-D-glucose	1.03	4.62	up
pmp001267	2-(Dodecylamino)-3-phenyl-1-propanol	1.21	2.01	up
pme3967	2-(Dimethylamino)guanosine	1.36	5.98	up
pmf0297	1-Eicosanol	1.38	2.08	up
Lmsn015919	Phytic acid	1.17	4.98	up
pme0026	L-Lysine	1.31	5.31	up
pmb0296	1-Oleoyl-Sn-Glycerol	1.39	3.79	up
Hmhp005160	Rehderianin I	1.37	3.09×10^{-1}	down
Lmgp004474	Genistein-7-O-galactoside-rhamnose	1.23	2.05	up
Lmyn005812	Gingerglycolipid A*	1.39	3.72	up
pmn001319	1-O-Feruloyl-3-O-p-Coumaroylglycerol	1.25	2.02	up
Lmyn006221	Gingerglycolipid C	1.39	3.76	up
Cmhp005227	4,8-Dihydroxy Naphthol-1-O-glucoside	1.01	4.03	up
pme3337	Succinyladenosine	1.35	3.05	up
pmb0736	Tricin-7-O-Glucoside*	1.22	2.25	up
Hmxp007521	3',4'-Dihydroxy-7,5'-dimethoxyflavone*	1.30	2.04	up
Hmqn003054	9,10,11-Trihydroxy-12-octadecenoic acid	1.38	2.39	up
Lmbn004240	9,10-Dihydroxy-12,13-epoxyoctadecanoic acid	1.36	2.04	up
Lmhp009384	1-Linoleoylglycerol-2,3-di-O-glucoside*	1.39	2.64	up
pmb0801	4-Pyridoxic acid-O-glucoside	1.38	3.69×10^{-1}	down
Lmbp002309	Sinapaldehyde-4-O-Glucoside	1.33	2.33	up
MWSmce632	Vanillin acetate	1.38	2.00	up
pmb0782	Piperidine	1.32	2.86	up
Lmhp009890	LysoPC 20:3	1.39	4.84	up
Lmxp000939	Zarzissine	1.33	2.31	up
MWS1882	Iminodiacetic acid	1.36	2.36	up
pmb1912	10-Formyltetrahydrofolic Acid	1.10	2.13	up
MWS2984	8-Azaguanine	1.22	2.16	up
Lmcp002302	N6-(2-Hydroxyethyl)adenosine	1.36	7.20	up
MWSmce607	DL-Threonine	1.32	2.07	up
MWSHY0141	7-Hydroxy-4-chromone	1.24	2.29	up
pmb2786	9-Hydroxy-10,12,15-octadecatrienoic acid	1.33	2.04	up
pmc0274	6-Methylmercaptopurine	1.35	3.45	up
MWS0205	Allantoin	1.30	3.69	up
pme0500	D-Melezitose	1.25	3.74×10^{-1}	down
Hmbp003234	1-O-Caffeoylglycerol	1.32	2.16	up
Zmhp004065	7,8-Dihydroxy-5,6,4'-trimethoxyflavone*	1.39	3.99×10^{2}	up
Rfmb320	1-Methylpiperidine-2-carboxylic acid	1.36	2.16	up
pme0243	Glutaric acid	1.39	3.75	up
pme0122	N6-Acetyl-L-lysine	1.37	3.41	up
Zmzn000113	L-threo-3-Methylaspartate	1.31	2.05	up
MWSmce295	1-beta-D-Arabinofuranosyluracil	1.29	2.27	up
pme0040	Adenine	1.27	2.25	up
mws1401	L-Theanine	1.29	3.31	up
pme0278	2,6-Diaminooimelic acid	1.36	4.57	up
Zmhn001446	Syringaresinol-4'-O-glucoside	1.23	8.08×10	up
pma2172	Cinnamoyltyramine	1.28	3.95×10^{-1}	down
Jmcp010061	Costic acid	1.29	4.34×10^{-1}	down
pme3388	Homoarginine	1.28	2.09	up
Lmqn008975	PA(18:2/0:0)	1.08	6.43	up
mws0668	Xanthosine	1.30	2.08	up
mws0866	D-Glucose 6-phosphate*	1.07	7.09	up
mws0133	Nicotinamide	1.38	5.22	up
pme3968	7-Methylguanine*	1.22	2.60	up
pmb1312	Tricin-4'-O-(guaiacylglycerol)ether-7-O-glucoside	1.27	3.27	up
Cmbn007148	1-O-Feruloyl-3-O-caffeoylglycerol	1.33	4.20	up

pme0193	L-Glutamine	1.34	3.46	up
Zmyn000453	Isocitric Acid*	1.35	2.55	up
pme2773	L-Cystathionine	1.16	3.49	up
pmb0490	p-Coumaroylputrescine	1.38	6.75	up
Jmbn003691	3,4-dihydroxy-allylbenzene-3-O-β-D-glucopyranoside	1.35	2.19	up
Hmhp001812	2'-O-Methyladenosine	1.32	2.83	up
Hmcp000405	Dihydroisopelletierine	1.20	1.06×10	up
MWSslk060	Eupatilin (5,7-Dihydroxy-3',4',6-Trimethoxyflavone)	1.00	3.67	up
Lmsn009824	2α,3β,19α,23-Tetrahydroxyolean-12-en-28-oic acid	1.31	3.82×10^{-1}	down
MWSmce333	Crotonoside; 2-Hydroxyadenosine	1.37	2.44	up
Zmpn000638	3-Guanidinopropionic acid	1.20	2.69	up
Lmhp008513	2-α-Linolenoyl-glycerol-1,3-di-O-glucoside*	1.33	3.30	up
mws0671	L-Homoserine	1.32	2.02	up
mws0258	L-Isoleucine*	1.35	2.17	up
mws0473	2-Methylsuccinic acid	1.34	3.94	up
Lmmn000806	Dimethylmalonic acid	1.34	3.94	up
Lmmn002164	Monomethyl succinate	1.34	3.94	up
Lmhp002031	L-Leucyl-L-Leucine	1.05	2.38	up
Jmcp009989	Pterodontic acid	1.36	4.47×10^{-1}	down
MWS2442	D-Fructose 6-Phosphate*	1.02	3.87	up
pmn001380	Eucommiol	1.37	2.40	up
Lmzp004885	Tricin (5,7,4'-Trihydroxy-3',5'-dimethoxyflavone)	1.36	3.47×10^{-1}	down
Xmzp009846	α-Ergocryptine	1.13	1.02×10	up
Lmgp004539	5,7,4'-Trihydroxy-6,8-dimethoxyisoflavone-7-O-galactoside-glucoside	1.19	2.13	up
pme3034	Ethylmalonic acid	1.32	2.77	up
pme1173	Allopurinol	1.34	3.12	up
Smpn009074	2α,3α,19α,23-tetrahydroxy-12-ursen-28-oic acid	1.40	7.64×10^{-4}	down
pme0010	L-Serine	1.36	2.54	up
pme2433	Diethanolamine	1.36	2.51	up
zjzp121501	Eupatorinol	1.28	2.44	up
pmb0891	Cis-Zeatin-7-N-glucoside*	1.28	4.90×10^{-1}	down
Zmjp004852	5,6,3',4'-Tetrahydroxy-3,7-dimethoxyflavone-6-O-glucoside	1.04	4.74×10^{-1}	down
Hmtp000776	4,5,6-Trihydroxy-2-cyclohexen-1-ylideneacetonitrile	1.31	3.65	up
pme2693	N-Acetylputrescine	1.31	4.99	up
Lmgp003546	4',5'-Dihydroxy-6,8-dimethoxyisoflavone-7-O-galactoside	1.12	2.43	up
Lmqp010784	Progesterone	1.40	8.95×10^{-4}	down
Lmhp008744	1-α-Linolenoyl-glycerol-2,3-di-O-glucoside*	1.32	3.63	up
pma3649	5-Aminolevulinic Acid	1.05	2.00	up
Jmgn005927	2-hydroxynaringenin	1.33	3.07	up
Lhhp120823	Eugenyl formate	1.28	2.57	up
pme0057	L-Homocysteine	1.03	4.10	up
pmb0496	N-Feruloylagmatine	1.39	5.79	up
pmb0764	4-Methyl-5-thiazoleethanol	1.08	2.04	up
mws0520	N-Acetyl-L-tyrosine	1.11	3.92×10^{-1}	down
Zmyn000247	2-Hydroxyglutaric Acid	1.05	6.19	up
Lmbn000216	3-Methylmalic acid	1.05	6.19	up
pme1286	S-(5'-Adenosy)-L-homocysteine	1.14	2.03	up
Lmjp003231	Patuletin-3-O-glucoside	1.10	2.22	up
Lmyp002912	2-Hydroxy-7-methoxy-1,4-benzoxazin-3(2H)-one (HMBOA)	1.29	3.11×10^{-1}	down
mws1589	D-Panose	1.25	3.94×10^{-1}	down
pmn001712	3-Hydroxy-4-isopropylbenzylalcohol-3-O-sophoroside	1.36	3.95×10^{-1}	down
mws0248	Uridine	1.13	2.22	up
Zmzp000145	Trimethyllysine	1.09	2.32	up
MWSmce498	Apigenin-7-O-neohesperidoside (Rhoifolin)*	1.10	2.24	up
mws0005	Tryptamine	1.34	3.32	up
pmb2857	L-Glutamic acid-O-glycoside	1.12	2.01	up
Lmmn002179	Methyl salicylate-2-O-glucoside	1.30	9.66	up
Lmhp008273	LysoPE 15:1(2n isomer)*	1.05	4.93	up

mws0889	D-Threonic Acid	1.20	2.70	up
Lmmp002013	Dihydroferuloylputrescine	1.38	1.49×10	up
pma1751	N-(beta-D-Glucosyl)nicotinate	1.26	2.42	up
Ymjm000143	2,3,4-Trihydroxybutyl 6-O-(E)-caffeoyl-β-D-glucopyranoside	1.27	4.40	up
Lmtp002942	Apigenin-6,8-di-C-arabinoside	1.01	2.15	up
mws1060	9-(Arabinosyl)hypoxanthine	1.24	4.81	up
Hmjp003400	1,7-Bis-(4-hydroxyphenyl)-2,4,6-heptatrienone	1.27	2.46	up
pme0368	Apigenin-7-O-rutinoside (Isorhoifolin)*	1.30	2.84	up
Lmdn006025	2-Hydroxy-2,3-dihydrogenistein	1.10	1.11×10	up
pmb0752	3-O-Feruloylquinic acid	1.24	2.93	up
Lmbp002255	Fraxetin-8-O-glucoside (Fraxin)	1.06	2.12	up
MWS4354	N6-methyladenosine	1.26	2.55	up
pmb3031	Tricin-4'-O-glycerol	1.09	1.35×10^{-1}	down
Lmpp003930	Apigenin-7-O-(6''-p-Coumaryl)glucoside	1.21	2.02	up
pmb0738	Tricin-7-O-(2''-Sinapoyl)glucoside	1.07	7.07	up

Table S7. The list of DAMs in ZH58×CH72/BFL-2.

Index	Compounds	VIP	Fold_Change	Type
Lssp210092	N-trans-ferulic tyramine*	1.59	2.58×10^{-1}	down
pmb0500	N-p-Coumaroyl-N'-feruloylputrescine	1.62	4.98×10^{-1}	down
Hmqp002567	4-Hydroxycinnamic acid p-hydroxyphenethylamine	1.54	9.88×10^{-2}	down
MWS0552	Cis-10-Pentadecenoic Acid(C15: 1)	1.38	2.26×10^{-1}	down
MWStz221	N-Cis-Feruloyltyramine*	1.56	2.57×10^{-1}	down
Zmyn004449	9-Hydroxy-12-oxo-10(E),15(Z)-octadecadienoic acid	1.64	1.22×10^{-1}	down
Hmbp006861	1,2-O-Diferuloylglycerol*	1.54	3.04	up
pmp000091	1,3-O-Diferuloylglycerol*	1.54	3.04	up
pme1014	Menatetrenone (Vitamin K2)	1.20	5.03	up
HJN092	p-Coumaroylferuloylputrescine	1.58	4.82×10^{-1}	down
MWSmce083	Ferulic acid methyl ester	1.63	1.71×10^{-1}	down
pme1184	2'-Deoxyguanosine	1.51	4.21×10^{-1}	down
MWSmce089	p-Coumaroyltyramine*	1.64	1.13×10^{-1}	down
pmn001320	1-O-p-Cumaroylglycerol	1.60	4.69×10^{-1}	down
mws0918	Prunetin (5,4'-Dihydroxy-7-methoxyisoflavone)	1.52	4.38×10^{-1}	down
pmb2789	13S-Hydroperoxy-6Z,9Z,11E-octadecatrienoic acid	1.16	2.86	up
Zmwp005562	cis-N-p-Coumaroyltyramine*	1.63	1.21×10^{-1}	down
Lmbn004685	5S,8R-DiHODE; (5S,8R,9Z,12Z)-5,8-Dihydroxyoctadeca-9,12-dienoate	1.65	3.74×10^{3}	up
pme2914	3-Hydroxy-3-methylpentane-1,5-dioic acid	1.12	4.03×10^{-1}	down
pme1841	Cadaverine	1.60	4.36×10^{-1}	down
pmp001281	LysoPC 18:1*	1.15	2.17	up
Lmlp003161	N-Feruloylputrescine	1.56	2.41×10^{-1}	down
mws1433	N-Feruloyltyramine*	1.60	2.62×10^{-1}	down
pme3011	γ-Aminobutyric acid	1.55	3.93×10^{-1}	down
Hmsp000364	L-Cyclopentylglycine	1.60	3.30×10^{-1}	down
MWStz294	N-Feruloyltryptamine	1.64	6.11×10^{-2}	down
pmn001319	1-O-Feruloyl-3-O-p-Coumaroylglycerol	1.45	2.42	up
MWSmce157	Stachydrine	1.63	2.03×10^{-1}	down
Jmzn006005	3,4-Methylenedioxy cinnamyl alcohol	1.63	1.25×10^{-1}	down
mws1200	Trans-4-Hydroxycinnamic Acid Methyl Ester	1.62	9.81×10^{-2}	down
pme2237	Dulcitol*	1.59	4.41×10^{-1}	down
pme3033	N,N-Dimethylglycine	1.51	4.81×10^{-1}	down
MWSHY0141	7-Hydroxy-4-chromone	1.35	4.18×10^{-1}	down
Lmhp009034	LysoPE 16:1*	1.65	4.17×10^{3}	up
Lmhp009464	LysoPE 17:1(2n isomer)*	1.58	4.85×10^{-1}	down
Hmbp003234	1-O-Caffeoylglycerol	1.61	3.50×10^{-1}	down
mws0214	D-Sorbitol*	1.60	3.81×10^{-1}	down
MWStz202	cis-Moschamine*	1.45	9.07×10^{-3}	down
Rfmb320	1-Methylpiperidine-2-carboxylic acid	1.63	1.94×10^{-1}	down

pme0122	N6-Acetyl-L-lysine	1.35	4.04×10^{-1}	down
MWSHY0118	5,6,7,4'-Tetramethoxyflavone	1.11	4.41×10^{-1}	down
MA10074217	4-Hydroxyquinoline	1.50	2.53	up
Cmmn013378	Levopimaric acid*	1.16	2.92	up
mws0133	Nicotinamide	1.59	4.12×10^{-1}	down
pme3968	7-Methylguanine*	1.22	2.26×10^{-1}	down
pmb1312	Tricin-4'-O-(guaiacylglycerol)ether-7-O-glucoside	1.45	4.34×10^{-1}	down
pmf0470	cis-4-Hydroxy-D-proline*	1.15	3.84×10^{-1}	down
pmb0962	L-Lysine-Butanoic Acid	1.61	3.33×10^{-1}	down
pme2773	L-Cystathionine	1.53	2.09×10^{-1}	down
Jmbn003691	3,4-dihydroxy-allylbenzene-3-O-β-D-glucopyranoside	1.58	4.93×10^{-1}	down
Hmcp000405	Dihydroisopelletierine	1.59	2.00×10^{-1}	down
Lmsn009824	2α,3β,19α,23-Tetrahydroxyolean-12-en-28-oic acid	1.52	2.99×10^{-1}	down
Lmbp000123	L-Homomethionine	1.20	4.37	up
Zmpn000638	3-Guanidinopropionic acid	1.13	2.38	up
mws1155	D-Mannitol*	1.47	4.13×10^{-1}	down
Lmgp008453	Albanin A	1.65	1.31×10^{-4}	down
pme1173	Allopurinol	1.49	4.53×10^{-1}	down
Smpn009074	2α,3α,19α,23-tetrahydroxy-12-ursen-28-oic acid	1.65	6.86×10^{-4}	down
Lmqp000873	Succinic anhydride	1.16	2.94×10^{-1}	down
pmb0770	N-Feruloylserotonin*	1.64	3.35×10^{-2}	down
pmb2795	4-Methoxycinnamic acid	1.63	1.01×10^{-1}	down
mws1195	p-Coumaric acid methyl ester	1.62	1.13×10^{-1}	down
zjgp122326	Pyridine-4-formyl-O-β-D-glucopyranoside	1.39	5.75	up
pme2693	N-Acetylputrescine	1.23	1.69×10^{-1}	down
pmb2855	L-Glutamine-O-glycoside	1.64	2.30×10^{-3}	down
pme0120	5-Aminovaleric acid	1.16	3.85×10^{-1}	down
MWS5209	N-Methyl-α-aminoisobutyric acid	1.61	3.46×10^{-1}	down
Jmgn005927	2-hydroxynaringenin	1.54	2.04	up
pmn001686	10,16-Dihydroxypalmitic acid*	1.17	2.66×10^{-1}	down
mws0520	N-Acetyl-L-tyrosine	1.23	1.58×10^{-1}	down
Lmsp008264	4'-Hydroxy-5,6,7-trimethoxyflavone*	1.54	3.60×10^{-1}	down
pme1286	S-(5'-Adenosy)-L-homocysteine	1.35	4.62×10^{-1}	down
mws0914	3,5,7-Trihydroxyflavanone (Pinobanksin)	1.21	2.15×10^{-1}	down
mws0943	1,4-Dihydro-1-Methyl-4-oxo-3-pyridinecarboxamide	1.45	3.07×10^{-1}	down
Hmxp006586	5-Hydroxy-7,3',4'-trimethoxyflavone*	1.51	4.22×10^{-1}	down
MWS4305	Erythrose	1.12	4.97×10^{-1}	down
pma0633	N-p-Coumaroylspermine	1.45	2.38×10	up
mws0005	Tryptamine	1.23	2.99×10^{-1}	down
Lmmp002013	Dihydroferuloylputrescine	1.64	3.07×10^{-3}	down
pma1751	N-(beta-D-Glucosyl)nicotinate	1.13	4.38×10^{-1}	down
pmb3107	Glucosyringic Acid	1.44	8.02×10^{-2}	down
pmb2620	3,4-Dimethoxycinnamic acid	1.63	1.46×10^{-1}	down
pme1654	Jasmonic acid	1.38	1.62×10^{-1}	down
pmb3075	3-O-p-Coumaroylshikimic acid	1.16	4.71×10^{-1}	up
Lmdn006025	2-Hydroxy-2,3-dihydrogenistein	1.48	2.23	down
Zmdp001857	γ-Glutamyltyrosine	1.23	4.33×10^{-1}	up
mws0437	D-Arabitol	1.38	3.95×10^{-1}	up
mws0213	Ribitol	1.38	3.95×10^{-1}	up
